# ﻿*Polygonatum dabieshanense* (Asparagaceae), a new species from the Dabieshan Mountains, Anhui and Henan provinces, China

**DOI:** 10.3897/phytokeys.269.173145

**Published:** 2026-01-06

**Authors:** Xiang-wen Song, Tao Xu, Wei Wang, Bang-xing Han, Guang-yan Li, Shan-yong Yi

**Affiliations:** 1 Traditional Chinese Medicine Institute of Anhui Dabie Mountain, West Anhui University, Luʼan, 237012, Anhui, China; 2 Anhui Engineering Research Center for Eco-agriculture of Traditional Chinese Medicine, West Anhui University, Luʼan, 237012, Anhui, China; 3 State Key Laboratory for Quality Ensurance and Sustainable Use of Dao-di Herbs, Beijing, 100700, China; 4 School of Pharmacy, West Anhui Health Vocational College, Lu’an, 237005, Anhui, China

**Keywords:** Asparagaceae, Dabieshan, *Polygonatum dabieshanense*

## Abstract

*Polygonatum dabieshanense* (Asparagaceae), a new species from the Dabieshan Mountains, Anhui and Henan provinces, China, is described and illustrated. It is most similar to *P. praecox*, *P. cyrtonema*, and *P. odoratum* but can be distinguished by having a yellow terete rhizome, a terete stem, elliptic leaf blades, racemose inflorescences, 1–2 (occasionally 3) flowers, a cylindrical perianth extending inwardly, filaments inserted near the distal 1/2 of the perianth tube that are smooth and glabrous, a white corolla, and blue-black berries, 0.8–1.0 cm in diameter, with 2–4 seeds. The complete chloroplast genome of the sampled individuals of this new species that we sequenced ranged from 155,107–155,108 bp in length. Our phylogenetic analyses, based on chloroplast sequences, confirm that the new species is distinct from morphologically similar species.

## ﻿Introduction

The genus *Polygonatum* Mill. (Asparagaceae: Polygonateae) is the largest genus within its tribe (Tang 1978; Chen and Tamura 2000). Plants of the genus *Polygonatum* are widely distributed across the northern temperate, northern subtropical, and frigid zones, with the highest diversity observed from the Himalayas to northern East Asia (Meng et al. 2014; Wang et al. 2016; Xia et al. 2022). Globally, there are over 80 species, of which more than 40 species are native to China, making it the center of distribution and diversification (Yang et al. 2022). In Traditional Chinese Medicine, Polygonati Rhizoma is derived from the dried rhizomes of several species of *Polygonatum* (Chinese Pharmacopoeia Commission 2020). Polygonati Rhizoma serves both as a medicinal herb and a food, possessing properties that tonify qi, nourish yin, strengthen the spleen, lubricate the lungs, and benefit the kidneys. Furthermore, over 20 additional species of *Polygonatum* are used medicinally in various regions (Zhao et al. 2022). Therefore, *Polygonatum* represents an important group of medicinal plants with high medicinal and edible value.

*Polygonatum* has received great attention from plant taxonomists, and its classification has been controversial. The most commonly used characteristics to distinguish species within *Polygonatum* include leaf arrangement, filament thickness, flower size, shape, and color, leaf type, and bract size and texture, among others (Baker 1875; Tamura et al. 1997; Chen and Tamura 2000; Meng et al. 2014; Wang et al. 2016). Additionally, molecular identification methods have been extensively applied in the taxonomic identification of *Polygonatum*. For instance, Tamura employed PCR amplification of the chloroplast gene *trnK* fragment to analyze the phylogenetic relationships among 14 species and one variety of *Polygonatum*, three species of *Disporopsis* Hance, and one species of *Heteropolygonatum* M.N.Tamura & Ogisu. The results were consistent with earlier taxonomic treatments and supported the separation of *Polygonatum* from *Disporopsis* and *Heteropolygonatum* (Tamura et al. 1997). With the advent of next-generation high-throughput sequencing technology, whole plastome sequencing has become cheaper and more efficient than ever, leading to the generation of an increasing amount of chloroplast genome data. Xia et al. (2022) conducted maximum likelihood (ML) and Bayesian inference (BI) analyses using 36 plastome sequences from 20 *Polygonatum* species and two *Heteropolygonatum* species, as well as 57 plastomes from 52 other outgroup species of Asparagaceae downloaded from GenBank (Xia et al. 2022; Floden and Schilling 2018). The results strongly supported the monophyly of *Polygonatum* and its sister relationship with *Heteropolygonatum*.

During surveys of wild germplasm resources of *Polygonatum* in eastern China, we collected several specimens of a plant that appeared to represent an undescribed taxon. These plants are characterized by alternate leaves, terete rhizomes, and greenish-white flowers measuring 1.5–2.0 cm in length. The species closely resembles *P. odoratum* and is often misidentified as that species in the field. However, it differs from *P. odoratum* in several morphological and phenological traits, including filament position (inserted near the distal one-half of the perianth tube, glabrous, and lacking apical saccate swellings), flowering period (early March to early April), and inflorescence type (racemose). Based on detailed morphological and phenological observations, chloroplast genome sequencing, and phylogenetic analyses, we confirmed that this taxon represents a previously overlooked species. Accordingly, we report the results of our investigations and herein describe and illustrate this new species, *Polygonatum dabieshanense* sp. nov.

## ﻿Materials and methods

### ﻿Population sampling

**Figure 1.**
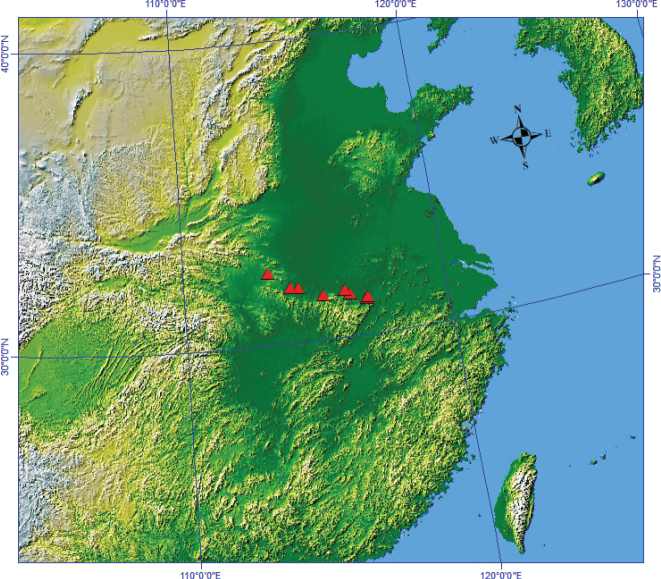
Distribution map of *Polygonatum dabieshanense*.

The new species was sampled at four locations during key developmental stages, including initiation of growth (February), flowering (March–April), and fruiting (April–October) in 2022 (Fig. 1; Table 1). A total of 65 individuals representing 47 *Polygonatum* species were included in this study, with eight individuals sampled for the new species. Chloroplast DNA (cpDNA) sequences of the new species were generated through sequencing, whereas cpDNA sequences of other species were obtained from the NCBI database. In addition, cpDNA sequences of two *Heteropolygonatum* species, *H. alternicirrhosum* and *H. ogisui* (accession numbers NC_058552 and NC_058553), were selected as outgroups.

**Table 1.** Basic characteristics of chloroplast genomes of *Polygonatum dabieshanense*.

Species name	Collection site	Voucher number	GenBank number
*Polygonatum dabieshanense*	Longmensi, Tongcheng, Anqing City, Anhui Province, China	Song 1DBSHJ	
*P. dabieshanense*	Nanyueshan, Huoshan, Luan city, Anhui Province, China	Song 2DBSHJ	PQ776918
*P. dabieshanense*	Jigongshan, Shihe, Xinyang City, Henan Province, China	Song 3DBSHJ	
*P. dabieshanense*	Tongbaishan, Tongbai, Nanyang City, Henan Province, China	Song 4DBSHJ	PQ776919
*P. dabieshanense*	Shimenchong, Shucheng, Luan City, Anhui Province, China	Song 5DBSHJ	
*P. dabieshanense*	Hongshigu, Jinzhai, Luan City, Anhui Province, China	Song 6DBSHJ	
*P. dabieshanense*	Huangbaishan, Shangcheng, Xinyang City, Henan Province, China	Song 7DBSHJ	
*P. dabieshanense*	Xiaojilongshan, Luoshan, Xinyang City, Henan Province, China	Song 8DBSHJ	

### ﻿Genome sequencing, assembly, and annotation

Two genomic DNA samples of the newly discovered species were extracted from silica-gel-dried leaves using the Tiangen DNAsecure Plant Kit (DP320). After extraction, whole-genome sequencing was performed on the BGISEQ–500 sequencing platform by Hefei Biodata Biotechnologies Inc. The chloroplast genome of *Polygonatum dabieshanense* was filtered and assembled using fastp (Chen et al. 2018) and SPAdes version 3.10.0 (Bankevich et al. 2012), respectively. Annotation of the chloroplast DNA (cpDNA) genome of *P. dabieshanense* was carried out using GeSeq (Tillich et al. 2017) and BLASTx (Gish and States 1993). The cpDNA sequences of *P. dabieshanense* were deposited in GenBank under accession numbers PQ776919 and PQ776918. The plastid genome map was generated using OGDRAW (Greiner et al. 2019). Basic characteristics of the chloroplast genomes of *P. dabieshanense*, *P. praecox* Ying Feng Hu & J.W.Shao, *P. cyrtonema* Hua, and *P. odoratum* (Mill.) Druce were analyzed and visualized in Geneious (Table 2).

**Table 2.** Basic characteristics of cpDNAs of *P. dabieshanense*, *P. praecox*, *P. cyrtonema*, and *P. odoratum*.

Characteristic	*P. dabieshanense*	*P. praecox*	*P. cyrtonema*	*P. odoratum*
Total length (bp)	155107–155108	155,121–155,256	155512–155816	154468
GC%	37.7%–37.7%	37.7%–37.7%	37.7%–37.8%	37.8%
LSC length (bp)	84016–84017	84,017–85,225	84618–84462	83417
SSC length (bp)	18445	18,458–18,474	18292–18440	18457
IR length (bp)	26323	26,318–26,323	26379	26297
Total genes	132	132	132	132
Protein-coding genes	86	86	86	86
rRNA genes	8	8	8	8
tRNA genes	38	38	38	38

### ﻿Phylogenetic analysis

To elucidate the phylogenetic relationships of the putative new species and its related taxa, plastome sequence data for *Polygonatum* species, including more than eight chloroplast genomes of *P. odoratum* publicly available in GenBank, together with those of the outgroup species *Heteropolygonatum alternicirrhosum* (Hand.-Mazz.) Floden and *H. ogisui*, were downloaded from GenBank. A preliminary maximum likelihood (ML) phylogenetic tree (Suppl. material 1) was constructed using FastTree version 2.1.10 under the GTR+GAMMA substitution model. Node support was evaluated using 1,000 site-likelihood resamplings to calculate local support values, and reliability was further assessed using the Shimodaira–Hasegawa (SH) test (Price et al. 2010). The preliminary ML analysis revealed that *P. odoratum* samples did not form a monophyletic clade. Instead, some samples clustered with *P. caulialatum*, others with *P. falcatum* A.Gray and *P. infundiflorum* Y.S.Kim, B.U.Oh & C.G.Jang, and one with *P. multiflorum* (L.) All. This pattern of polyphyly is most likely attributable to misidentification of voucher specimens, as *P. odoratum* should phylogenetically group only with *P. caulialatum* S.R.Yi.

To address this issue, a rigorous sampling-screening procedure was implemented. First, wild *P. odoratum* specimens were re-sampled across their natural distribution range in China, subjected to detailed morphological verification following the diagnostic characters provided by Chen and Tamura (2000), and sequenced for their chloroplast genomes. The resulting sequence was deposited in GenBank under accession number PX404798 and used as a taxonomic reference standard. Second, all *P. odoratum* plastome sequences obtained from GenBank were re-evaluated, and only those that clustered with the reference *P. odoratum* sequence (PX404798) in the preliminary ML tree were retained. Samples showing ambiguous or conflicting phylogenetic placements were excluded from further analyses.

The final phylogenetic analysis was conducted using a curated dataset comprising 65 individuals representing 47 *Polygonatum* species, including two individuals of the new species, verified *P. odoratum* samples, and representatives of morphologically similar species such as *P. praecox* and *P. cyrtonema*. *Heteropolygonatum alternicirrhosum* and *H. ogisui* M.N.Tamura & J.M.Xu were designated as outgroups. The ML tree was reconstructed using the same methodology described above. To further confirm the robustness of the inferred phylogenetic relationships, a BI tree was also generated using MrBayes v3.2.7 (Ronquist et al. 2012). Bayesian analyses were performed using Markov chain Monte Carlo (MCMC) sampling for 100,000 generations. The nucleotide substitution model GTR+F+I+G4, previously selected using ModelFinder, was applied in the BI analysis.

## ﻿Results and discussion

**Figure 2.**
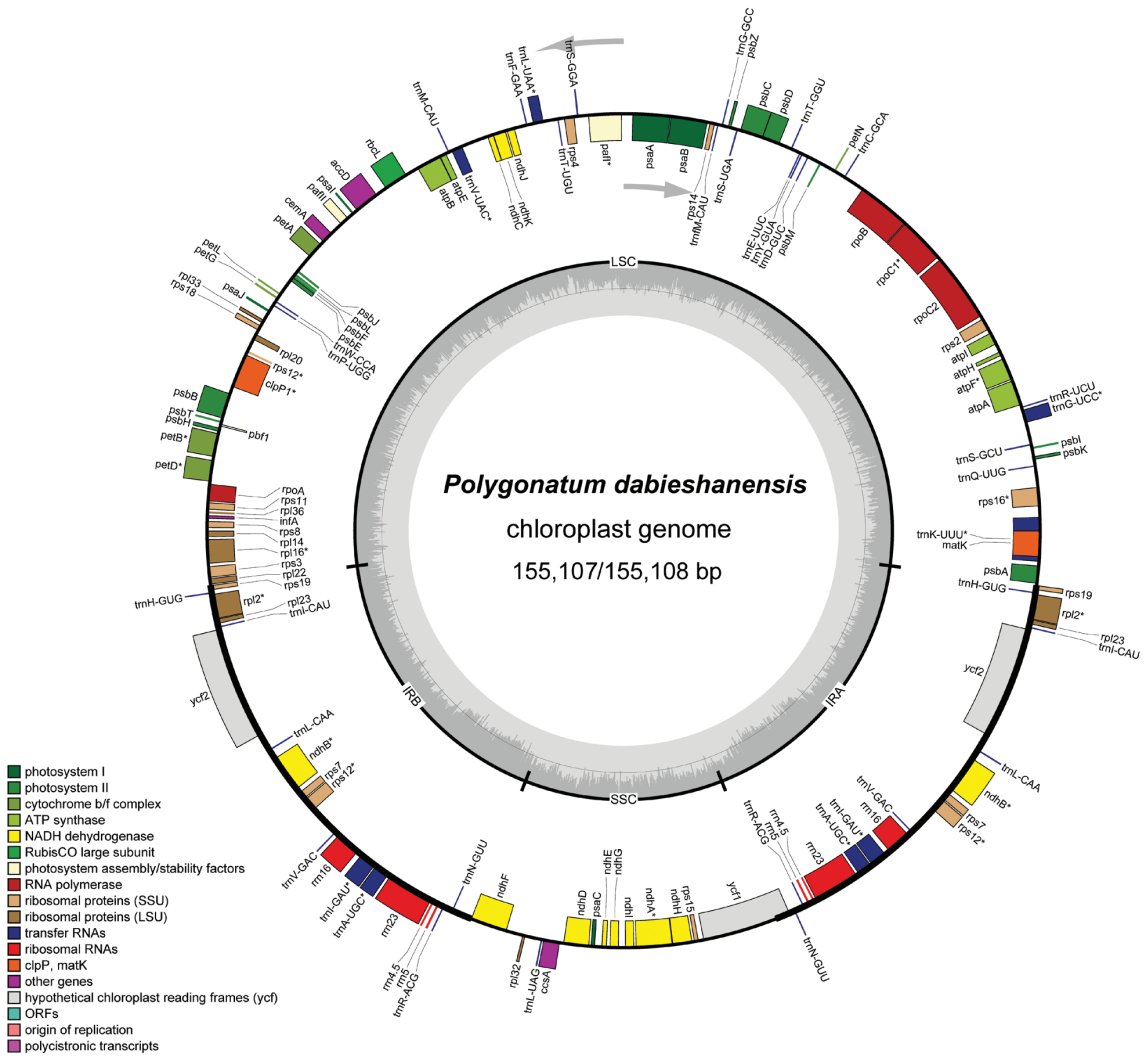
Plastid genome map of *P. dabieshanense* B.X.Han,S.Y.Yi & X.W.Song, sp. nov.

The complete chloroplast genome sequences of the four samples of *Polygonatum dabieshanense* ranged from 155,107 to 155,108 bp in length (Fig. 2), whereas those of *Polygonatum praecox* ranged from 155,115 to 155,256 bp (Hu et al. 2022). Both species possessed a typical quadripartite structure, comprising inverted repeat region A (IRa), inverted repeat region B (IRb), a large single-copy region (LSC), and a small single-copy region (SSC). A comprehensive summary of the distinctive features and statistical analyses of their cpDNAs is presented in Table 2.

**Figure 3.**
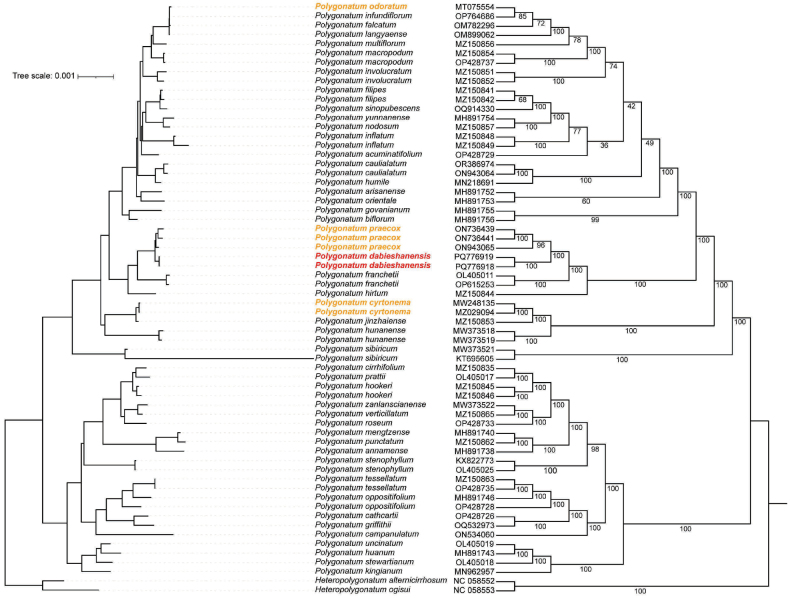
Phylogenetic relationships of the new species and its related species in *Polygonatum* based on complete chloroplast genomes. It includes the maximum likelihood tree (ML tree, left) and Bayesian inference tree (BI tree, right) constructed using 47 representative species, with *H. alternicirrhosum* and *H. ogisui* as outgroup taxa. Numbers on the branches indicate bootstrap support values from the ML and BI analyses. The phylogenetic position of *P. dabieshanense* is highlighted in pink, *P. praecox* in red, and *P. odoratum* in blue. GenBank accession numbers are shown after the species names.

The two specimens of the putatively new species, originating from two distinct populations in different provinces, were clustered into a monophyletic clade with robust bootstrap support (BS) of 100%. Notably, this emerging species exhibited a sister-taxon relationship with *P. praecox*, also supported by a BS value of 100%. Despite this phylogenetic proximity, substantial morphological divergence, particularly in rhizome characters, was observed between the new species and *P. praecox* (Table 3). Intriguingly, the new species did not share a sister-taxon relationship with the other two morphologically similar species, namely *P. odoratum* and *P. caulialatum* (Fig. 3). Furthermore, although the new species displayed the closest morphological resemblance to *P. odoratum*, a significant genetic divergence was observed between them in terms of evolutionary relationships.

**Table 3.** Comparison of *P. dabieshanense*, *P. praecox*, *P. cyrtonema*, and *P. odoratum*.

Characters	*P. dabieshanense*	*P. praecox*	*P. cyrtonema*	*P. odoratum*
Rhizome	terete, 1.0–2.0 cm thick, yellow	moniliform,1.6–2.8 cm thick, yellow	gingeriform, 1.5–3.0 cm thick, yellow	terete, 0.5–1.0 cm thick, white
Stem	30–70 cm, terete	30–85 cm, terete	40–105 cm, terete	25–65 cm, upper part angled
Leaves	blade elliptic	blade oblong-lanceolate	blade oblong-lanceolate	blade elliptic
Inflorescence	raceme, 1–2(–3) flowered	raceme, 1–3(–4) flowered	umbel-like, 3–7(–14) flowered	raceme, 1–3(–5) flowered
Filament	cylindrical and extending inwardly, smooth, and glabrous	cylindrical and extending inwardly, smooth, and glabrous	papillose or shortly cottony, apex slightly dilated or saccate-convex	cylindrical and extending inwardly, smooth or verruculose
Inserted position of filament	filaments inserted near the distal 1/2 of the perianth tube	filaments inserted near the distal 1/3 of the perianth tube	filaments inserted near the distal 1/3 of the perianth tube	filaments inserted near the distal 1/2 of the perianth tube
Corolla	white, lobes excurved	green, lobes excurved	green, lobes excurved	white, lobes slightly excurved
Berries	blue-black, 0.8–1.0 cm	black, 1.2–1.5 cm	black, 0.9–1.0 cm	blue-black, 0.7–1.0 cm
Seed	2–4	9–15	3–9	7–9
Flower phenology	March to April	March to April	April to May	March to April

### ﻿Taxonomic treatment

#### ﻿ Polygonatum dabieshanense

Taxon classificationPlantaeAsparagalesAsparagaceae

B.X.Han, S.Y.Yi & X.W.Song sp. nov.

D484BDD6-BD82-5839-8861-41C3271B284B

urn:lsid:ipni.org:names:77374666-1

Figs 4, 5

##### Diagnosis.

The new species similar to *P. praecox*, *P. cyrtonema*, and *P. odoratum*. It differs from *P. praecox* in having a terete rhizome, white corolla, 2–4 seeds; differs from *P. cyrtonema* in having a terete rhizome, white corolla, filament cylindrical and extending inwardly, smooth, and glabrous, 2–4 seeds; differs from *P. odoratum* in having a yellow rhizome, upper part angled stem, 2–4 seeds.

**Figure 4.**
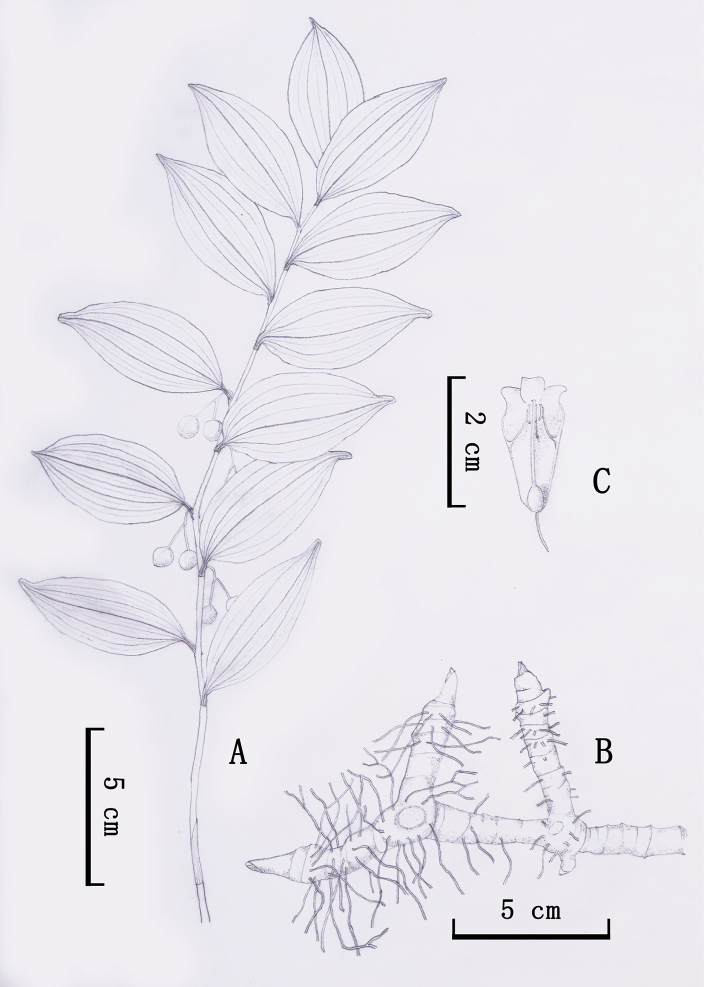
*Polygonatum dabieshanense* B.X.Han, S.Y.Yi & X.W.Song, sp. nov. **A.** Habit; **B.** Rhizome; **C.** Flower. Drawn by Tao Xu.

##### Type.

China • Anhui Province: Luan City, Huoshan County, Mt. Nanyueshan, 12 Mar 2022, *Song SXW220312* (holotype: ACM; isotype: PE).

**Figure 5.**
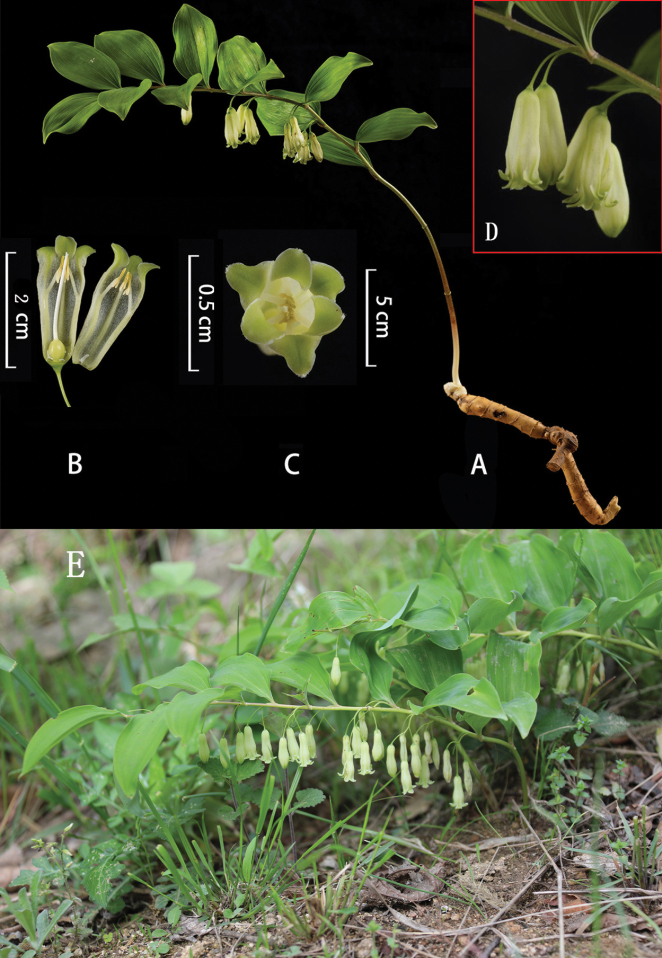
*Polygonatum dabieshanense* B.X.Han, S.Y.Yi & X.W.Song, sp. nov. **A.** Plants; **B, C.** Flowers; **D.** Inflorescence; **E.** Habitat. Photos by Tao Xu.

##### Description.

Rhizome terete, 1.0–2.0 cm thick. Stem arching, 30–70 cm, glabrous, and terete. Leaves 10–18, alternate; petiole short or nearly sessile; leaf blade elliptic, 5.0–7.0 × 3.5–5.0 cm, apex usually short mucro. Inflorescences raceme, 1–2(3) flowered; peduncle 1.0–1.5 cm; bracteoles borne on the middle part of pedicel, subulate, 0.5–1.5 mm, or absent. Flowers pendulous, pedicel 0.5–1.0 cm long. Perianth white, campanulate-cylindrical, 1.5–2.0 cm long; lobes 2–3 mm long, excurved. Filaments inserted near the distal 1/2 of the perianth tube, cylindrical and extending inwardly, 2.5–5.0 mm long, smooth, apex without saccate-convex. Anthers 3.0–3.5 mm long. Ovary 3–5 mm in diam. Berries blue-black, 0.8–1.0 cm in diam, 2–4 seeded.

##### Phenology.

Growth initiated in February, flowering in March–April, and fruiting in April–October.

##### Distribution and habitat.

Primarily in moist, deciduous, bamboo forests and broad-leaved forests, 150–1000 meters; documented on the slopes of Dabieshan Mountains, in Anhui and Henan Provinces.

##### Etymology.

Derived from the name of the Dabieshan Mountains, where *Polygonatum dabieshanense* was discovered.

##### Chinese name.

大别山黄精 (da bie shan huang jing)

##### Morphological comparison.

Morphological comparisons between the new taxon and other *Polygonatum* species revealed that it is most similar to *P. praecox*, *P. cyrtonema*, and *P. odoratum*. However, it can be distinguished from *P. praecox*, *P. cyrtonema*, and *P. odoratum* by distinct features of the rhizome, leaves, stem, inflorescence, filament, corolla, and flowering phenology (Table 3).

## Supplementary Material

XML Treatment for Polygonatum dabieshanense

## ﻿Additional information

### Use of AI

No use of AI was reported.

### Author ORCIDs

Xiang-wen Song https://orcid.org/0009-0001-3356-6428

Tao Xu https://orcid.org/0009-0004-9155-3651

Wei Wang https://orcid.org/0000-0003-3100-6184

Bang-xing Han https://orcid.org/0000-0002-1696-6104

Guang-yan Li https://orcid.org/0009-0007-0395-508X

Shan-yong Yi https://orcid.org/0000-0001-6653-1515

### Data availability

All data that support the findings of this study are available in the main text, Supplementary Information or in the NCBI database (https://www.ncbi.nlm.nih.gov/).

## Supplementary materials

Supplementary material 1 Phylogenetic tree Data type pdf Explanation note A preliminary phylogenetic tree constructed with representative chloroplast genomes of all reported *Polygonatum* species (with partial representatives selected for species with multiple available plastomes). https://binary.pensoft.net/file/1506174This dataset is made available under the Open Database License (http://opendatacommons.org/licenses/odbl/1.0/). The Open Database License (ODbL) is a license agreement intended to allow users to freely share, modify, and use this Dataset while maintaining this same freedom for others, provided that the original source and author(s) are credited.Xiang-wen Song, Tao Xu, Wei Wang, Bang-xing Han, Guang-yan Li, Shan-yong Yi

Supplementary material 2 Chloroplast intergenic spacers Data type pdf Explanation note Two chloroplast intergenic spacers (*psb*K-*psb*I (A) and *psb*A-*trn*H (B)) from *Polygonatum robustum* Nakai compared with homologous sequences of *P. dabieshanense*. https://binary.pensoft.net/file/1506175This dataset is made available under the Open Database License (http://opendatacommons.org/licenses/odbl/1.0/). The Open Database License (ODbL) is a license agreement intended to allow users to freely share, modify, and use this Dataset while maintaining this same freedom for others, provided that the original source and author(s) are credited.Xiang-wen Song, Tao Xu, Wei Wang, Bang-xing Han, Guang-yan Li, Shan-yong Yi

Supplementary material 3 *Polygonatum simizui* Kitag. and *Polygonatum dabieshanense* Data type docx https://binary.pensoft.net/file/1506176This dataset is made available under the Open Database License (http://opendatacommons.org/licenses/odbl/1.0/). The Open Database License (ODbL) is a license agreement intended to allow users to freely share, modify, and use this Dataset while maintaining this same freedom for others, provided that the original source and author(s) are credited.Xiang-wen Song, Tao Xu, Wei Wang, Bang-xing Han, Guang-yan Li, Shan-yong Yi

Supplementary material 4 *Polygonatum robustum* and *Polygonatum dabieshanense* Data type docx https://binary.pensoft.net/file/1506177This dataset is made available under the Open Database License (http://opendatacommons.org/licenses/odbl/1.0/). The Open Database License (ODbL) is a license agreement intended to allow users to freely share, modify, and use this Dataset while maintaining this same freedom for others, provided that the original source and author(s) are credited.Xiang-wen Song, Tao Xu, Wei Wang, Bang-xing Han, Guang-yan Li, Shan-yong Yi
