# Supplementary material for: ﻿Polygonatum dabieshanense (Asparagaceae), a new species from the Dabieshan Mountains, Anhui and Henan provinces, China
Source: PhytoKeys. 2026 Jan 6;269:31–42. doi: 10.3897/phytokeys.269.173145 (PMC12800783; doi:10.3897/phytokeys.269.173145)
Supplement: Supplementary material 1 — Phylogenetic tree [file phytokeys-269-031_article-173145__-s001.pdf]

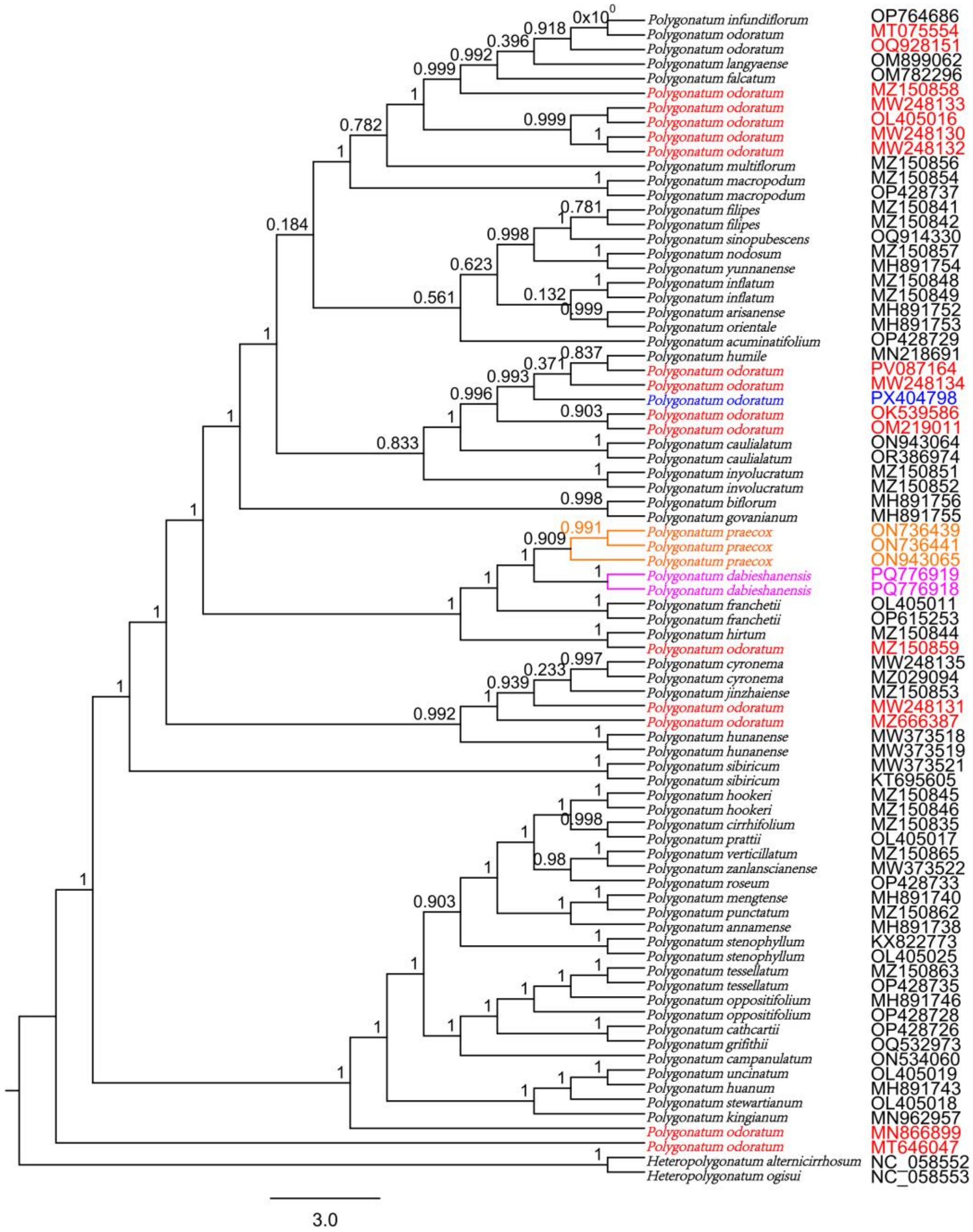

Figure S1 A preliminary phylogenetic tree constructed with representative chloroplast genomes of all reported *Polygonatum* species (with partial representatives selected for species with multiple available plastomes)
