# Supplementary material for: ﻿Polygonatum dabieshanense (Asparagaceae), a new species from the Dabieshan Mountains, Anhui and Henan provinces, China
Source: PhytoKeys. 2026 Jan 6;269:31–42. doi: 10.3897/phytokeys.269.173145 (PMC12800783; doi:10.3897/phytokeys.269.173145)
Supplement: Supplementary material 2 — Chloroplast intergenic spacers [file phytokeys-269-031_article-173145__-s002.pdf]

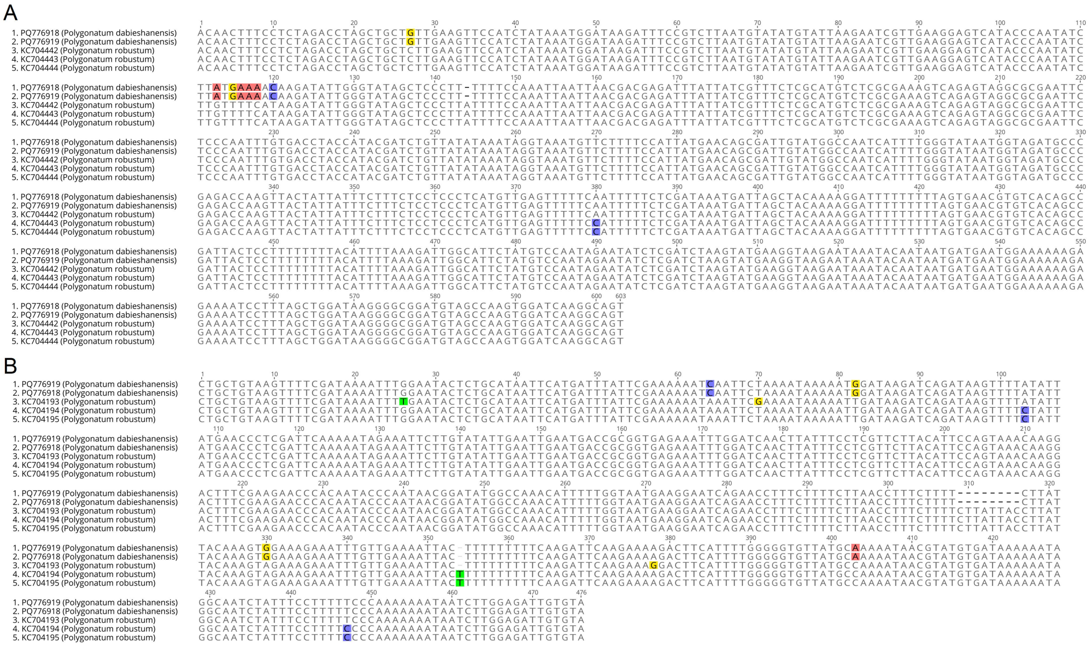

Figure S2 Two chloroplast intergenic spacers (*psb*K-*psb*I (A) and *psb*A-*trn*H (B)) from *Polygonatum robustum* compared with homologous sequences of *P. dabieshanensis*.
