# Supplementary figures and images for: ﻿Polygonatum dabieshanense (Asparagaceae), a new species from the Dabieshan Mountains, Anhui and Henan provinces, China
Source: PhytoKeys. 2026 Jan 6;269:31–42. doi: 10.3897/phytokeys.269.173145 (PMC12800783; doi:10.3897/phytokeys.269.173145)

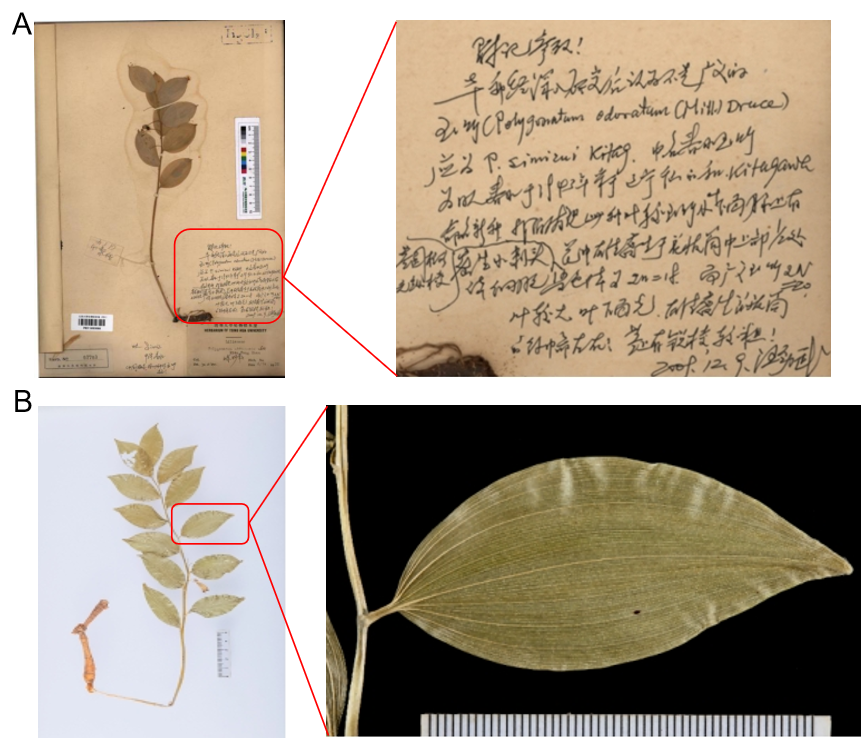


Fig. S3 A: *Polygonatum simizui* andB: *Polygonatum dabieshanensis*

Supplement: Supplementary material 3 — Polygonatum simizui Kitag. and Polygonatum dabieshanense [file phytokeys-269-031_article-173145__-s003.docx]

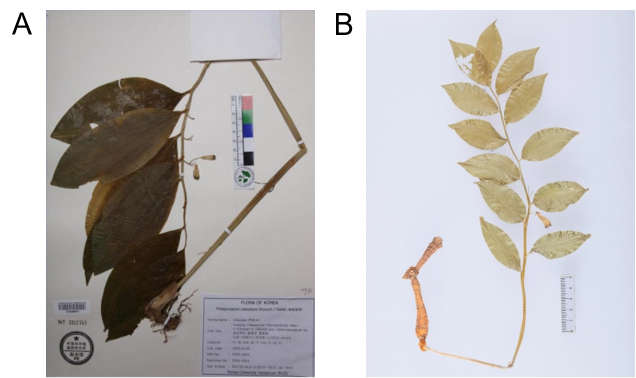


Fig. S4 A: *Polygonatum robustum* andB: *Polygonatum dabieshanensis*

Supplement: Supplementary material 4 — Polygonatum robustum and Polygonatum dabieshanense [file phytokeys-269-031_article-173145__-s004.docx]
